# Evolution of Nursing Education, Research and Practice: Insights to Foster Nurse Solidarity and Address Future Challenges

**DOI:** 10.1111/jan.70449

**Published:** 2026-01-03

**Authors:** William H. C. Li

**Affiliations:** ^1^ The Nethersole School of Nursing, Faculty of Medicine, the Chinese University of Hong Kong Hong Kong China

## 1 Introduction

In celebration of the 50th anniversary of the *Journal of Advanced Nursing*, this commentary explores the evolution of nursing education, research and practice over the past five decades. In particular, we reflect on the key milestones, transformative innovations and pivotal developments that have shaped the profession, particularly during a period marked by rapid technological advancement, the growing burden of noncommunicable chronic diseases (NCDs), the global impact of the coronavirus disease 2019 (COVID‐19) pandemic, and the challenges posed by ageing populations.

Amid these transformations, the role of nurses has become increasingly vital. In every setting, our adaptability, resilience and commitment have been instrumental in maintaining the stability of the healthcare system. This commentary discusses how these four key milestones have shaped the nursing profession, influenced patient‐centred care, developed nursing practices and nursing knowledge and increased the impact of nursing on global healthcare systems.

This commentary will highlight the pivotal research, policy changes, and transformative shifts in nursing education and practice over the past half century and identify the major challenges that the nursing profession is likely to face over the coming decades. Central to this forward‐looking perspective is the concept of solidarity—defined as unity and mutual support within a group—which we propose as a guiding principle for addressing future challenges in nursing. By fostering solidarity among nurses, healthcare professionals, and communities, we can strengthen our collective capacity to navigate the evolving landscape of global health.

## 2 Evolution of Nursing Education, Research and Practice Over the Past Five Decades

Nursing education, research and practice have undergone revolutionary changes following important milestones over the past 50 years, which have reshaped the nursing profession. The first part of this commentary delves into four pivotal areas: the transition to higher education, shifts towards specialisation and advanced practice, the impacts of pandemics, and an ageing population. Taken together, these milestones reflect the dynamic and progressive nature of nursing and underscore its vital role in healthcare.

### 2.1 Transition to Higher Education

Until the mid‐20th century, nurse training was primarily based on hospital apprenticeships focusing on practical, task‐oriented skill development. In the past five decades, the transition from traditional apprenticeship‐based training models to university‐based education has created the foundation for a well‐educated nursing workforce capable of managing the complexities of modern healthcare. In the 1980s, a transformative wave in university‐based nursing education began in the United States and soon spread to other countries. This progression came in response to increasingly sophisticated healthcare needs that required a more educated workforce to meet the higher complexity of the healthcare system. In 1983, the Institute of Medicine in the US called for the creation of a national centre for nursing research and recommended that nursing research be integrated into practice to improve healthcare outcomes, in addition to increasing the numbers of university‐educated nurses (National Institute of Nursing Research 2025). The report also suggests a new transformative wave to promote the value of teaching nursing in higher education. Around 1990, a key nursing initiative, Project 2000: A New Preparation for Practice, was launched in the UK to support the teaching of nursing, midwifery and health visiting practices throughout the educational system (Le Var 1997). Following Project 2000, the former apprenticeship model was replaced by baccalaureate and higher degree programmes in university‐based education. Similar transitions were adopted in other Western countries, such as Canada and Australia.

In Hong Kong, tertiary‐level nursing education dates to the late 19th century, with its longstanding emphasis on high‐quality foundational training and postgraduate specialisation. Over the years, the region has made significant strides in advancing nursing education, culminating in the establishment of the Bachelor of Nursing degree as the standard qualification for registered nurses. This shift reflects global trends towards university‐based curricula and the professionalisation of nursing. Meanwhile, Mainland China—home to the world's largest nursing workforce—continues to expand its healthcare capacity and elevate educational standards through coordinated national initiatives (Wu 2023).

This transformation from vocational training to formal nursing education in universities has marked the growing global solidarity in nursing education. Organisations such as the International Council of Nurses and the World Health Organisation (WHO) have facilitated knowledge sharing through publishing guidelines (e.g., WHO's Global Standards for Professional Nursing Education), enabling low‐resource countries to adapt university‐based models. Thus, the transition to university‐based nursing education was successful. In April 2023, the results of the 2022 National Nursing Workforce Survey showed that the proportion of registered nurses with a bachelor's degree or higher in the US workforce exceeded 70% for the first time (American Association of Colleges of Nursing 2024). This is close to the Institute of Medicine's (2011) recommendation that 80% of the nursing workforce hold a bachelor's degree by 2020. Today, nurses are increasingly pursuing doctoral degrees (van Dongen et al. 2024), which will have a significant impact on future nursing care and patient outcomes. Most importantly, this transformation in nursing education has paved the way for nursing specialisation and advanced practice nursing (APN).

### 2.2 Transition to Specialisation and Advanced Practice Nursing

As healthcare demands have evolved, the roles of nurses have also changed, resulting in the emergence of advanced practice nurses with specialised skills and knowledge (Bryant‐Lukosius et al. 2004). These advanced practice nurses are expected to acquire higher and broader levels of competencies to assume specialised roles in the healthcare system.

The concept of APN first emerged in the United States and Canada in the 1960s and gradually gained international recognition. The UK adopted this nursing concept during the 1970s, and it later spread to other countries. In 1994, the Hospital Authority of Hong Kong introduced the nurse specialist role, which was then reclassified in 2000 as an advanced practice nurse role. In 2009, the ranking position of nurse consultants was introduced to systematically enhance the impact of APN professionals in the healthcare system.

Advanced practice nurses play multiple roles in the healthcare system, including providing evidence‐based practice, leading education, performing consultations, initiating service development, supervising continuous quality assurance, and conducting research. Apart from their advanced clinical practice duties, they are also expected to play crucial roles in evidence‐based research to inform nursing practices and healthcare reforms.

Master's programmes in Nursing initially focused on advanced general practice. Over time, these postgraduate programmes began to offer supervised practice in speciality fields. Advanced practice nurses now serve to bridge healthcare disciplines, fostering interdisciplinary solidarity to improve patient outcomes, which further promotes the development of nursing specialisation and APN. Establishing institutional frameworks is essential to support and formalise these evolving roles.

In 2011, the Hong Kong Academy of Nursing was established and immediately played an important role in creating systems to support APN. In particular, a statutory certification system was established to protect the safety of the public and to legitimise APN. In 2024, the institution was renamed the Hong Kong Academy of Nursing and Midwifery. Hong Kong's APN model aligns with global trends emphasising advanced clinical skills, leadership and specialist roles within nursing career progression. About 5000 advanced practice nurses are currently working in inpatient and specialised settings in Hong Kong, demonstrating competencies comparable to those of their international counterparts.

### 2.3 Impacts of Pandemics: A Resilient and Informed Nursing Profession

Over the past 50 years, the global healthcare system has confronted two major pandemics, severe acute respiratory syndrome (SARS) and COVID‐19, each leading to significant changes in the evolution of nursing practices and education. The SARS experience has prepared the nursing workforce for public health emergencies by strengthening infection control practices and crisis response strategies. For example, Thompson et al. (2004) advocated that nursing curricula be revised to strengthen the education of all healthcare professionals. The SARS pandemic also revealed the potential for innovation in nursing education, as technological advancements and the new knowledge produced by research and continuing education have played pivotal roles in the development of professional competencies in APN, including in leadership, organisational change, and evidence‐based practice (Wright et al. 2024).

Moreover, the COVID‐19 pandemic had a profound impact on the mental well‐being of the population. The sudden and overwhelming demand for skilled healthcare providers revealed both the resilience of nursing professionals and the vital importance of maintaining a robust, adaptable nursing workforce. This experience underscored the importance of preparing nurses not only with clinical expertise but also with emotional and psychological support to give them resilience to weather the high‐pressure challenges of providing healthcare during global pandemics. In particular, offering psychological support for nursing professionals to protect their ongoing well‐being within the complex requirements of modern healthcare environments became of paramount importance during the unprecedented challenges of the COVID‐19 pandemic (Cheung et al. 2023). Hence, we now understand the importance of early interventions, such as providing individual and organisational support for nurses, including flexible scheduling and rest periods, to ensure the future readiness of the healthcare system. Consequently, the COVID‐19 pandemic provided a pivotal moment for nurse practitioners and policymakers to reassess best practices in the healthcare system, including the safety and quality of patient care, in addition to their own needs (Smith et al. 2020).

Nurse educators have also played crucial roles following the disruption of nursing education during the COVID‐19 pandemic (Ion et al. 2021). In particular, nursing education quickly shifted from face‐to‐face teaching to adopting technological advancements in online and hybrid learning models to ensure that nursing students could continue their education despite lockdowns and social distancing measures during the pandemic. However, the impact of in‐person clinical training was undermined. In response to this issue, nurse educators employed innovative teaching strategies to provide simulation‐ and virtual reality‐based education to allow their students to complete their nursing education. Up to 50% of the required clinical hours were replaced by simulations during the pandemic (Martin et al. 2023).

As more nursing students are now trained in universities, the COVID‐19 pandemic also highlights the need to adapt flexible educational methods due to the limited learning opportunities in hospitals following pandemic restrictions. Simulations and other innovative methods can become alternatives to the tight schedules required for clinical placements. However, there are some considerations to address when incorporating simulations as a permanent component of the nursing curriculum, such as balancing the advantages of traditional clinical placements against the allocation of resources, faculty development, and curriculum development to ensure that nursing students gain empathetic, human‐touch experiences with real patients. Additionally, the shift to online learning during the COVID‐19 pandemic imposed tremendous stress on nursing students, raising concerns about their limited clinical practice opportunities, restricted physical classroom interactions with classmates, and potential graduation delays.

From an organisational perspective, educational institutions must consider implementing strategies to support their future students' mental health and build resilience in cultivating their ability to manage the potential stressors of future pandemics. To enhance clinical reasoning skills both in school and at home, advanced technologies, such as online virtual simulations, have been adopted.

During the COVID‐19 pandemic, nursing researchers generated new knowledge to inform best practices. For example, they addressed immediate clinical challenges, such as the psychological impact of COVID‐19 on healthcare workers (Lorente et al. 2021). Studies conducted among advanced practice nurses described their expanded roles and responsibilities during the pandemic, especially in the direct care of patients with COVID‐19, individualised patient care and healthcare team support, which informed best practices and highlighted the importance of interdisciplinary and supportive environments (Chair et al. 2024).

Overall, the robust and rapid research output during the COVID‐19 pandemic has directly impacted and informed advanced practice nurses' future clinical practices, operational strategies, educational practices and policies (Choi et al. 2020). In particular, these new standards have laid the groundwork for an increasingly resilient and informed nursing profession in the future.

### 2.4 Ageing Population and Non‐Communicable Diseases

Over the past five decades, population ageing has emerged as a major global concern, with profound social, economic, and health‐related implications. According to the World Health Organization (WHO), global life expectancy at birth has risen from approximately 65 years in 1970 to over 73 years by 2024 (World Health Organization 2025). This demographic shift has significantly contributed to the rise in NCDs—including cardiovascular diseases, cancer, diabetes, and chronic respiratory conditions—which are now among the leading and largely preventable causes of morbidity and mortality worldwide (World Health Organization 2013).

The growing prevalence of NCDs among older adults has placed considerable strain on healthcare systems, resulting in increased patient loads, more complex care needs, and heightened staffing pressures. These challenges contribute to elevated levels of nurse burnout and workforce turnover. Nursing education has had to rapidly adapt by integrating gerontology, NCD management, and palliative care into curricula, often stretching institutional resources. The demand for specialised geriatric care continues to outpace the supply of trained nurses and qualified educators, particularly in ageing‐related fields. Moreover, the physical and emotional demands of elder care may deter students from pursuing careers in this area, further exacerbating workforce shortages.

## 3 Future Nursing Challenges

In considering the most significant developments, innovations and milestones in nursing education over the past half century in this commentary, we must ask what the key challenges that the nursing profession is likely to face over the next half century are and how we can address these challenges to meet our global population's future healthcare needs. In our ever‐changing world, providing healthcare services has never been so complex. Therefore, this third part of our commentary aims to illuminate different aspects of the current and future challenges for nursing education and practice, such as the transformation of nursing education, nurturing the younger generations of nurses, technological advances (especially the evolution of artificial intelligence [AI]), nursing shortages and professionalism, and the implications of an ageing population.

### 3.1 Transformation of Nursing Education

Following advances in healthcare technologies, increased medical knowledge, growing awareness of the public's right to be informed, greater emphasis on patient rights and safety, and higher public expectations of nursing services, nurses today face more challenges than ever before. Therefore, it is crucial to identify novel strategies to prepare new generations of nursing professionals to face these challenges and work effectively within increasingly complex healthcare delivery systems. Over half a century, nursing training in most parts of the world has undergone a major transition from hospital‐based nursing training to university‐based nursing education, with a bachelor's degree in nursing being the basic requirement for qualified registered nurses. However, many still question the necessity of college nursing education. Some even ask whether advanced nursing education can be directly translated into effective nursing practice to improve the quality of care and patient outcomes. Although we believe that university‐based nursing education is the optimal means of improving nursing services, more evidence is needed to defend this proposition (Coster et al. 2018). For example, a retrospective observational study of nurse staffing, educational attainment, and hospital mortality in 300 hospitals across nine European countries provided strong evidence that employing an adequate number of well‐educated nurses in acute care areas can reduce the risk of patient mortality (Aiken et al. 2014). Furthermore, there is evidence that both advanced and specialist nurses and physicians provide equally safe care to patients with chronic diseases and that advanced and specialist nurses are at least as effective as physicians in primary care management (Kane et al. 2007; Massimi et al. 2017). Therefore, we must continuously demonstrate to the public and reassure our clinical partners that high‐quality primary nursing education directly translates into effective daily nursing practices.

### 3.2 Nurturing the Younger Generations of Nurses

Nurturing and equipping the next generation of nurses to face and address global healthcare challenges over the next 50 years is of paramount importance. In addition to providing training to develop competent nurses with skills in interpersonal communication, analysis, problem‐solving and leadership, what else can we do to nurture new generations of nursing professionals? Nursing education must be reshaped in line with changing trends and needs to guide future developments in the nursing profession. Hence, nursing education curricula and advanced practice programmes should also be revised to improve methods for cultivating the critical thinking skills required for nurses to perform with higher levels of responsibility and accountability. Unfortunately, competition among various nursing education institutions around the world is fierce. These institutions expend most of their time and energy in pursuit of better resources and global rankings, in addition to raising entry‐level qualifications to attract young people to join the profession. Harry Lewis (2006), a professor at Harvard for more than 30 years and dean of Harvard College for 8 years, drew from his experience to write a book called *Excellence Without a Soul*, which provides educators of the current generation some good reflections on how reputed universities have abandoned their educational mission. In his book, Lewis (2006) reminds us that the fundamental goal of education is to help young people grow, learn who they are, and search for a greater purpose in their lives. Above all, higher education must transform young people into adults who are willing and able to take responsibility for society. In addition to emphasising the importance of achieving academic and clinical excellence, it is crucial to develop the next generation of nurses by equipping and empowering them to improve the world and by fostering their personal strength, integrity, kindness, co‐operation and compassion. In particular, cultivating a humanistic approach to nursing education will promote and sustain nurses' adherence to patient‐centred care and help them develop deeper insights into their own values and life experiences. Most importantly, this approach will foster humanitarian values, such as respect, solidarity, empathy and compassion, in new generations of nursing professionals.

### 3.3 Technological Advances in Nursing Education

Another challenge for nursing education is the need to develop innovative teaching pedagogies to meet the learning needs of new generations of nurses. While it is important to adopt innovative approaches in nursing education, the pursuit of emerging technologies while ignoring nursing students' substantive learning needs often wastes time and resources. Therefore, it is essential to identify the actual need for the adoption of innovative technologies in nursing education and to conduct research to evaluate and document the effectiveness of newly adopted teaching strategies in teaching and learning, particularly to identify whether the new approaches are better than previous ones. Rigorous research on nursing education is needed to generate evidence for best practices that can be used to guide the teaching process. Understanding how research and education impact the quality of healthcare and patient outcomes is essential for future investments and development in nursing education globally.

### 3.4 Evolution of Artificial Intelligence

The rapid development of AI in recent years has brought as many challenges as opportunities, with creative applications in many scientific fields, including nursing education. The use of AI‐powered simulators and virtual patients in nursing education has allowed nursing students to practice their clinical skills and decision‐making in safe environments. Using augmented, mixed, and virtual reality tools to create immersive and interactive educational environments can promote nursing students' engagement and improve their cognitive abilities and clinical performance.

Although AI tools have created many teaching breakthroughs, their credibility, privacy, ethics and integrity issues cannot be ignored (Michalowski et al. 2025). However, nursing educators must learn how to use novel technologies to advance their teaching practices and keep pace with their more technology‐savvy students. When instructing students to integrate AI technologies into their practices, nursing educators must ensure that these tools are used responsibly and emphasise that their students use their critical thinking and judgement to analyse the complex data generated.

### 3.5 Impact of New Technologies on Nursing Education

Brazilian educator Paulo Freire (2005) has emphasised that the most important thing about education is its ‘humanisation’; however, education is regretfully losing its humanising aspirations by relying solely on innovative technologies and has become pure training through the transfer of knowledge content (Freire 2005), which is no different from training animals. That is, education trains people to compromise with the world instead of cultivating their human intelligence (Freire 2005). In particular, human intelligence is vital for successful nursing education. Nursing educators have valuable clinical experience and expertise that can contextualise the output of AI applications and help students understand the differences in patient care beyond using AI algorithms. Most importantly, as ‘life influences life’, nursing educators must help their students discover their personal strengths, develop well‐rounded characters and be compassionate and empathetic to provide personalised patient care. In addition to ensuring their patients' safety, advanced practice nurses must respect their patients' autonomy and self‐esteem, empathise with their suffering and take action to alleviate their pain.

In summary, the synergy between AI tools and APN is vital, as AI can act as a support tool rather than as a replacement for the complex and deeply human aspects of nursing practice. In particular, humanity is an indispensable core value of nursing that AI cannot replace. As medical technologies continue to advance and AI continues to evolve, maintaining a balance between humanity and the benefits of AI is crucial for nursing education today and in the future.

### 3.6 Nursing Shortages and Professionalism

The growing global nursing shortage is currently the biggest problem facing the nursing industry and poses serious challenges for global healthcare systems. Increasing and strengthening the training of new generations of nurses is a feasible method for addressing this concern; however, governments and relevant authorities must increase their investments in nursing resources in higher education, introduce advanced teaching technologies and qualify nursing educators to improve their teaching quality. Improving nursing morale and establishing specialist nurse qualifications are the best methods for achieving this goal. Unfortunately, many countries have still not recognised the need for nursing professionalism. We firmly believe that investments in high‐quality nursing education and scientific research will greatly improve public health. We recommend that governments in other countries without a statutory registration system for specialist nurse qualifications must implement such a system as soon as possible, formulate a timetable for the legislative process and promote the development of APN. Additionally, researchers must assess how expanded nursing roles have long‐term impacts on morale, sense of accomplishment, job satisfaction, retention and professional development, which will thereby improve the quality of care and patient outcomes.

### 3.7 Impact of Ageing Population and Non‐Communicable Diseases

In the coming five decades, ageing populations and the rising prevalence of NCDs will continue to pose significant challenges for healthcare systems and nursing education, driving up healthcare expenditures and increasing the global socioeconomic burden. In Hong Kong, for example, the Hospital Authority projects a 50% increase in NCD cases among the middle‐aged population—from 2 million in 2019 to 3 million by 2039—threatening the sustainability and capacity of the healthcare system (Health Bureau, HKSAR 2023). In response, the government has committed to strengthening district‐based primary care, including the establishment of health centres in every district and the launch of the Chronic Disease Co‐Care Pilot Scheme in 2023 for adults aged 45 and above.

Looking ahead, prevention will be key. The adage ‘an ounce of prevention is worth a pound of cure’ is especially pertinent in public health. Adolescence represents a critical developmental stage marked by rapid biological and emotional changes and increased exposure to risk behaviours such as smoking, alcohol use, poor diet, and physical inactivity—all of which contribute to NCDs later in life. These trends underscore the importance of initiating health education and screening efforts at a younger age. As global healthcare models shift from treatment‐focused to prevention‐oriented approaches, early intervention during youth will be essential for promoting lifelong health and reducing the future burden of NCDs.

To effectively address the challenges posed by ageing populations and the growing burden of NCDs, healthcare systems must prioritise prevention and the long‐term management of chronic conditions. Nursing education should evolve to equip future nurses with the competencies required for geriatric care, NCD prevention and management, and interdisciplinary collaboration. Strategic reforms are essential, including the integration of age‐related health content across all levels of training, the expansion of clinical placements in community and elder care settings, and the incorporation of advanced technologies—such as simulation, telehealth, and artificial intelligence—to enrich teaching and learning. Furthermore, cultivating interest in geriatric nursing through mentorship programmes, career development incentives, and public awareness initiatives will be vital to building a resilient and responsive nursing workforce capable of meeting the demands of an ageing society.

## 4 Conclusions

In addition to the challenges discussed above, nurses will face unprecedented challenges in the next 50 years, such as climate change, extreme weather, natural disasters, ageing populations and epidemics, particularly the ongoing impact of the COVID‐19 pandemic, which cannot be ignored. These future challenges may have significant impacts on nursing education in the preparation of new generations of nurses. Hence, nurse educators should continue to review and update their curricula to equip these new generations with the skills to address these challenges and meet the future needs of our global population. Considering these huge challenges, we cannot act alone, nor can we rely on the strengths of individual countries to solve them. Nurses worldwide must stand in solidarity to address these global challenges.

Finally, as we face the challenges ahead, it may be worth looking at the example of how Florence Nightingale overcame many obstacles to becoming a nurse and note her key role in the development of the nursing profession. Nightingale regarded nursing as the job that most intimately dealt with people's illnesses and suffering (Pfettscher 2021). She believed that being able to take care of the sick and relieving their pain were not just a job but a divine task. Nightingale's contributions to the nursing profession and their relevance today can be illustrated by her promotion of comprehensive nursing education, which emphasises cutting‐edge science education and high ethical standards. In particular, Nightingale suggested that nurses must have independent thinking and analytical and judgement capabilities and should play a vital role in infection prevention, infection control, isolation, containment and public health (Pfettscher 2021). Even today, her dedication inspires nurses to continually improve their skills and remain fearless in the face of difficult challenges to advocate for the best healthcare for their patients.

## Funding

The author has nothing to report.

## Conflicts of Interest

The author declares no conflicts of interest.

## Data Availability

The data that support the findings of this study are available on request from the corresponding author. The data are not publicly available due to privacy or ethical restrictions.
